# The Study of Spatial Safety and Social Psychological Health Features of Deaf Children and Children with an Intellectual Disability in the Public School Environment Based on the Visual Access and Exposure (VAE) Model

**DOI:** 10.3390/ijerph18084322

**Published:** 2021-04-19

**Authors:** Ning Ma, Sa Ma, Shuangjin Li, Shuang Ma, Xinzhi Pan, Guohui Sun

**Affiliations:** 1 College of Art and Design, Beijing University of Technology, Beijing 100124, China; maning@bjut.edu.cn; 2 Shenzhen Key Laboratory of Spatial Information Smart Sensing and Services, School of Architecture and Urban Planning, Research Institute for Smart Cities, Shenzhen University, Shenzhen 518060, China; sama14@snu.ac.kr; 3 Graduate School for International Development and Cooperation, Hiroshima University, Higashi Hiroshima 739-8529, Japan; d196414@hiroshima-u.ac.jp; 4 Research Center for Advanced Science and Technology, The University of Tokyo, Tokyo 153-8904, Japan; 5 Laguardalow Architect, New York, NY 10041, USA; jimmy.pan@laguardalow.com; 6 Beijing Key Laboratory of Environment and Viral Oncology, Faculty of Environment and Life, College of Life Science and Chemistry, Beijing University of Technology, Beijing 100124, China

**Keywords:** visual accessibility, visual exposure, spatial safety, public psychological health, school architecture

## Abstract

Nowadays, there is increasing attention towards the safety and feelings of children in urban or architectural space. In this study, the authors suggest a new approach based on the Visual Access and Exposure (VAE) Model to evaluate the spatial safety and social psychological health features of deaf children and children with an intellectual disability in the public school environment. The authors present a preliminary study of deaf children and children with an intellectual disability in a primary school located in Deyang by measuring the visual exposure and visual access in the public environment. The results illustrate that there are a few spaces, such as a long corridor and the space behind the elevators, that are not very safe for deaf children and children with an intellectual disability. In terms of social psychosocial preference, this special group prefers to stay in low visual access areas, which may be influenced by their introverted and impaired social communication ability. This study could have implications for the existence and optimization of an architecture design for relevant groups. With the increase in school bullying incidents and public psychological health problems related to youth, this approach could be used widely in the area of school safety and public psychological health management.

## 1. Introduction

Currently, there is increasing attention towards children’s health and safety, as well as their feelings about the urban or architectural environment [1,2]. The visual analysis method is also widely used to evaluate spatial safety. However, few studies refer to deaf children and children with an intellectual disability, and there exist little effective records and research on buildings’ visual characteristics.

The VAE Model was proposed by Archea. This model indicates how the selection of one’s location within an architecturally bounded setting can affect both the acquisition of information about the surrounding activities and the ability of others to take notice of one’s own behavior. The VAE Model is used for analyzing the potential visual access or visual exposure degree. In this model, visual access (VA) refers to the degree to which information can be obtained from a point through unobstructed visual surveillance, and visual exposure (VE) is the degree to which an individual is visible from other destinations in the space [3,4,5]. According to Archea [3] and Choi [6,7,8], in terms of social behavior, the VA is equivalent to the locationally based opportunity to adjust one’s own behavior according to the possible existence and behavior of others in the surrounding spaces. Additionally, again in terms of social behavior, the VE is equivalent to the locationally generated degree of obligation or acceptability to conform to one’s own behavior according to the possible existence and behavior of others in the surrounding spaces.

There are several research methodologies about the visual presentation of urban and architectural forms. For example, Benedikt and other scholars defined a visual isovist that represents the visibility towards various directions, which measures the isovist of various space shapes and sizes and formulates a series of variables to evaluate people’s space perception [9,10]. Those isovist properties always include the total area, perimeter, vertices number and density, openness and roundness [11]. Based on these isovists, Turner et al. generated a set of visibility graphs to study the architectural space [12]. The Spatial Openness Index (SOI) exerts a 3D visibility and permeability analysis [13]. Additionally, Hillier et al. suggested a space syntax that refers to the visibility and accessibility in space [14,15].

However, most studies have focused on space morphology and configuration. The VAE model differs in the view shed, while isovist modeling specifically focuses on calculating how much of a surface feature can be seen. This model is concerned with architecture contributions to the regulations for routine social encounters when considering the visual permeability from both the VA and VE dimensions. In addition, this model is unlike the conventional models of environment and behavior, which mainly concentrate on actual human behavior and relate it to specific environment properties; in contrast, the VAE model treats the built environment as a place to structure spatial behavior [8]. In a special location, a high VA value means that the people in this space would have more opportunities to adjust their behavior by identifying the range of behaviors that are acceptable or appropriate within the prevailing social context [3]. Additionally, in a special location, a high VA value indicates that those in this space would receive more visual scrutiny by others, which could cause more normative pressure to align their behavior to the prevailing social context [8]. This means that observing the spatial configuration of the built environment can connect a human behavioral pattern with social psychological health considerations. Consistently, a preferred location is a space that is a more comfortable space or that conforms more to a person’s social psychological features (such as introverted or extroverted personality) to perform a related behavior. Another advantage of the VAE Model is that it can quantitatively measure the built environment.

Previously, the VAE Model was widely used in studies of the conducting of criminal acts and in evaluating the space quality [5,16]. For example, Dickey used the VAE Model as a framework to investigate bank robbers and suggested that bank robberies always occur in buildings that have architecture with special visual criteria [17]. Archea chose VA and VE for a warehouse, storefront, residence and record store in a selected site to explore the ways in which perpetrators of certain types of crime utilize the physical arrangement of settings to support their normative expectations and criminal intentions [5]. In An, Ko and Kim’s study, a quantitative calculation was made of the VE grade to determine the extent of the visual damage caused by residential development [18]. Bartie et al. introduced a new VE model to establish an observer’s true evaluation when passing through vegetated regions [19]. However, few studies refer to the safety of the public environment.

Based on the advantages of the VAE Model, in this article, the authors propose to apply the VAE as an effective approach to evaluate the physical safety and public psychological health features of deaf children and children with an intellectual disability, whose lives are mainly based on the visual features of a public space in a built environment, to lead the architectural design for this special group. There exist only a handful of studies based on the built environment and deaf children and children with intellectual disabilities. Anaby et al. studied the effect of the built environment on the participation of children and youth with intellectual disabilities through a literature review [20]. Fauconnier et al. suggested improving the accessibility to toilets, ramps, lifts, aids, parking and public transportation for those with intellectual disabilities to enhance their physical safety and social participation [21]. The size and layout of the buildings, as well as the crowds and distance, are also argued as being important built environmental attributes that should be considered in the design for children with an intellectual disability [22]. Colver et al. highlighted the importance of emotional and physical support from environmental items, through the European Child Environment Questionnaire, as well as from teachers [23]. However, in these studies, there is no empirical analysis to evaluate a detailed relationship.

Specifically, this paper has the following two study objectives. Firstly, this research will propose a method based on the visibility features of VA and VE in the VAE model to evaluate the physical safety of the public environment of the school architecture of a primary school in Deyang, focusing on deaf children and children with an intellectual disability. Secondly, through observing the spaces where deaf children and children with an intellectual disability prefer to stop and by checking the VA and VE features of these spaces, this paper will indicate the social psychological health features of deaf children and children with an intellectual disability.

Based on the study objectives, the major contributions of this study include the following aspects. Firstly, this research suggests using the VE degree in the VAE model in the public environment design of school architecture for the safety of deaf children and children with an intellectual disability. In addition, this research proposes using the VA degree in the VAE model to determine the socially isolated children, in order to provide them with more support for their psychological problems. By checking the VE value and the VA/VE value of the preferred staying space of individual children in a general school, this approach could be widely used to determine the places where potential bullying may occur and identify the students with possible psychological health problems.

## 2. Materials and Methods

### 2.1. Theoretical Background

#### 2.1.1. How Is the VA, VE Associated with Physical Safety for Deaf Children and Children with an Intellectual Disability?

Safety in the public environment means that spaces allow individuals to go about their daily lives without being uncomfortable or afraid of harm. The VAE degree is very important in preventing crime and ensuring spatial safety, and, according to Jane Jacobs, safety, particularly for women and children, comes from “eyes on the street,” i.e., the surveillance of public spaces [24,25,26]. This means that spaces with a high VE are safer for the general public.

Compared with the general public, the safety problems of disabled groups, especially deaf children and children with an intellectual disability, are always very serious [27]. The visual characteristics, mainly the high VE degree in space, are very important for the safety of this special kind of children. Disability children who have limited ability to move, see, hear or make decisions, as well as those who do not feel or understand pain, might not realize that something is unsafe, or might have trouble getting away. In addition, compared with their healthy peers, over 10% of children with a disability have experienced child abuse [28]. Thus, teachers should monitor the spaces for these children to ensure that children are always in a safe situation.

Therefore, the first purpose of this VAE model is to test the VE situation of the public environment for deaf children and children with an intellectual disability in a primary school in Deyang, and we suggest that a space with a high VE has greater physical safety.

#### 2.1.2. How Are the VA and VE Associated with the Psychological Health Features of Deaf Children and Children with an Intellectual Disability?

People spend most of their time indoors. This provides the setting in which we live, and it has an impact on people’s senses and emotions [29,30,31,32]. Staying in a too narrow space can lead to some people becoming anxious or short of breath, and they might feel faint or confused. Buildings with higher degrees of scaling and contrast are perceived as being more natural in psychological responses [29].

It is very crucial to translate the users’ psychological features and behaviors into the real architecture environment [30]. Altman, as early as 1974, linked the built environment, behaviors and environmental or social psychological features by conceptualizing “privacy” [33,34,35]. Forgas and Jones researched the social psychology of interactions in the public space, the sense of belonging and the preferred space of the general public [36]. Yadegari and Alinaghi studied the spatial usage difference between introverts and extroverts in an architectural public space [37]. According to Lawson, people sit in places where they can simply watch the scenery around them, representing an escapist community, while a concave area creates communication and socialization [38].

According to Archea, in the VAE model, instead of treating rooms and buildings as homogeneous environment entities, the spatial attributes, such as the VA and VE, are important variables in the built environment and are coordinated with human behavior [3,4,5]. In other words, the arrangement of the physical environment regulates the distribution of the information upon which all interpersonal behavior depends. In addition, observing human behavior (such as preferring a place to stay in) in a space and the spatial attributes, such as the VA and VE, can reflect the social psychology. This social psychology, which is a branch of psychology, is accepted as being an attempt to understand, and explain how, the thoughts, feelings and behaviors of individuals are influenced by the actual, imagined or implied presence of others [39,40]. Shyness, low self-esteem, feelings of stress in a group and social anxiety, as well as converse performance, are all social psychological features from the perspective of visual surveillance by their teachers or other people.

**Figure 1:**
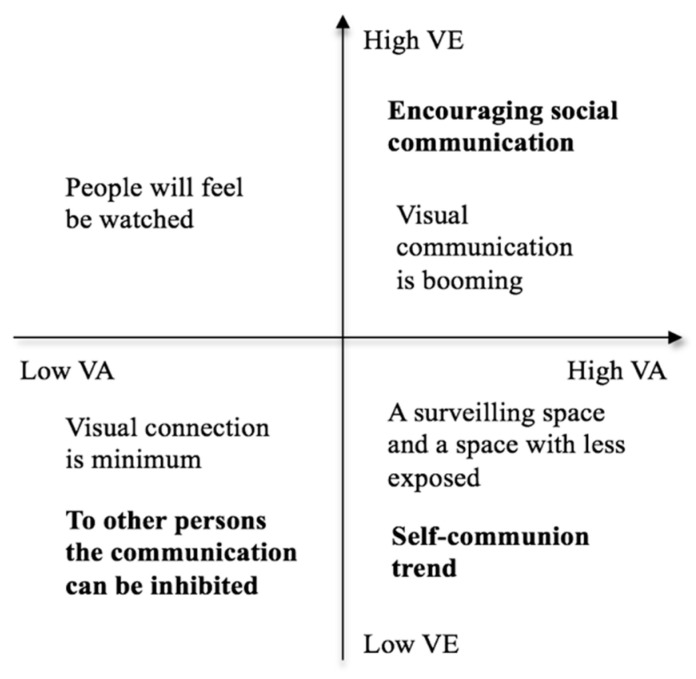
The relationship between VA, VE and psychological health features in the social community.

From an empirical point of view, in a place with a high VA and high VE, the visual communication is booming. This kind of place is always an open public environment that encourages social communication in buildings. In a place with neither a low VA nor a low VE, the visual connection is minimal. Due to the low VA, a visitor is denied access to the cues needed to determine the host’s receptiveness to intrusion and, with a low VE, to other persons, communication can be inhibited. It is a kind of place where shy and self-enclosed people tend to stay, as it does not cause claustrophobia. A high VA and a low VE form a surveillance space and a space that is less exposed. In contrast, people will feel they are being watched in a space with a low VA and a high VE. In their daily life, humans need various VAs and exposure spaces, either an open space with a high VA and VE to partake in social activities or a quiet space with low VE values for self-communion (Figure 1).

Thus, the second purpose of the VAE model is to observe the public environment that an individual child prefers to stay in and to check the VA and VE in this space. Additionally, the psychological health features of deaf children and children with an intellectual disability, in terms of social-communication preference, can be evaluated in the VAE model.

### 2.2. Study Area

The primary school in Deyang, Sichuan province that was investigated in this study is a charitable educational institution focused on deaf children and children with intellectual disabilities. The designers of this architecture at the China Southwest Architectural Design and Research Institute Co., Ltd. stated that this design could offer more opportunities for students to explore the world in public areas.

**Figure 2:**
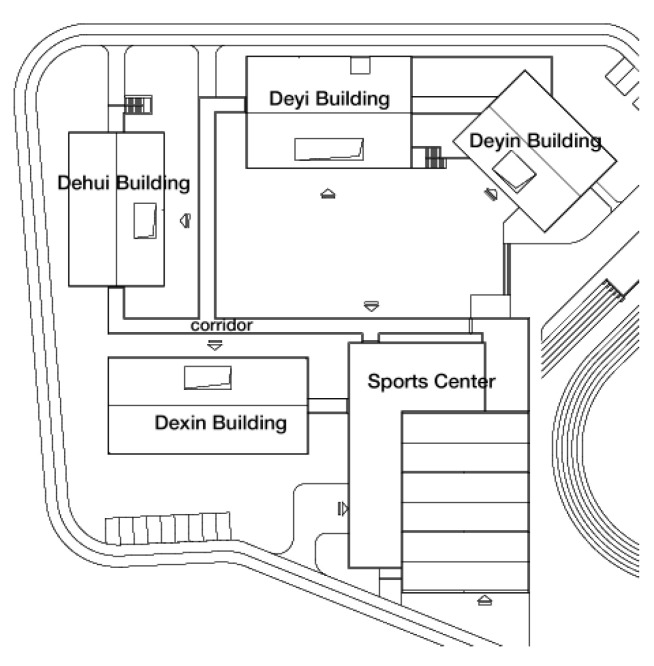
The scale floor plan of the special primary school in Deyang.

The data for this school in the research are provided by the China Southwest Architectural Design and Research Institute Co., Ltd., and includes detailed scale floor plans and construction drawings. This school consists of five individual buildings. These buildings surround a central courtyard and contain classrooms, rehabilitation therapy rooms, sports facilities, a library and accommodation for students. Two crossed corridors are used not only as rain canopies for children to move from one building to another, but also to offer interesting spaces (between close spaces and open spaces) for exploring and enjoying leisure time (Figure 2).

### 2.3. Methodology

#### 2.3.1. Evaluation of the VA and VE Features in the VAE Model for the Public Environment

**Figure 3:**
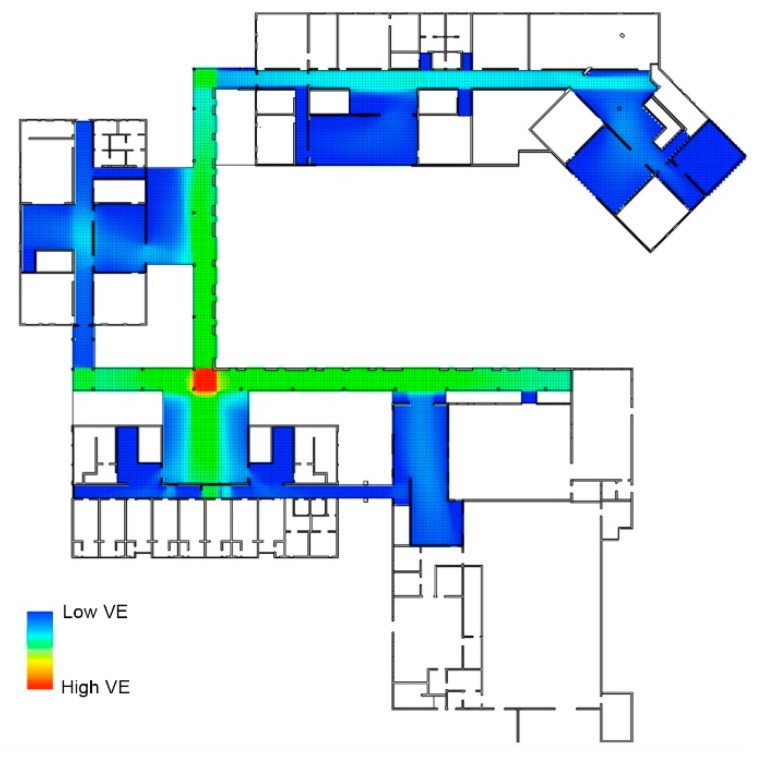
The distribution of the VE value in the special primary school.

This part of the research methodology is composed of the following steps:

- Simplifying the floor plan. In the first step, the floor plan was simplified with AutoCAD 2017. The authors removed all the furniture, ventilation shafts and electrical equipment and kept only the integrated walls of the public environment in the school buildings and the two crossed rain corridors/canopies. The authors removed the ventilation shafts and electrical equipment from the construction drawing, as they cannot impact the space usage in the public space. Certain amenities could be significant obstacles for the children. The school manger left some furniture in the public space, with the height of this furniture being lower than the eyesight of the primary school children. Thus, the locations of these amenities were not considered.
- Processing the windows and doors. The visual accessibility from the outside to the interior of the architecture was not considered in this study. The authors used the walls instead of the windows (and doors) for the exterior wall in AutoCAD 2017 and reserved the windows in the public environment. The authors removed the drawing of doors in AutoCAD 2017 in the floor plan of the public environment, in order to ensure that eyesight would be taken into account in the calculations.
- A set of grid points was created with 70 cm of distance. Through the former two steps, the enclosed public environment, including the indoor public space, two interaction canopies outside and their connecting space, can be created (Figure 3). Next, a set of grid points was generated according to the assumed spatial distance between people on the plans with OPERA software. In this study, the authors chose 70 cm as the assumed spatial distance between people.

This methodology is based on Choi’s previous research. The VA value at every point refers to the total number of grid points that can be seen as one looks from any given grid point to all of the other grids’ points in the built environment [6,7,8]. The VE values in every grid point refer to the total number of times each grid point is seen from each of the other grid points.

#### 2.3.2. Observing the Space Usage of the Public Environment for Deaf Children and Children with an Intellectual Disability

The field study method is used in this part of the research methodology as follows: the real public environment usage was observed for the two connected buildings, i.e., the Deyin and Deyi Buildings, at 10:10 during a morning in May 2017. The authors chose this time period because it is the start of break time between classes. The average age of the students in the Deyin and Deyi buildings is 10.5 years old. A total of 27 static children were observed in the public school environment. Two of them remained static to look at posters on the wall, while the other children remained static without being attracted to the surrounding amenities; therefore, the spatial locations of only 25 children were considered. This study defines the children as static when they stayed in one place for more than two min (over 20% of the total break time).

Communication could be a core topic of social psychology and the purpose of the field study observation is to compare the space usage and the visual preference of the public environment to evaluate the psychological health features of deaf children and children with an intellectual disability in social communication.

## 3. Results

### 3.1. The VA, VE and Physical Safety in the School Environment

**Figure 4:**
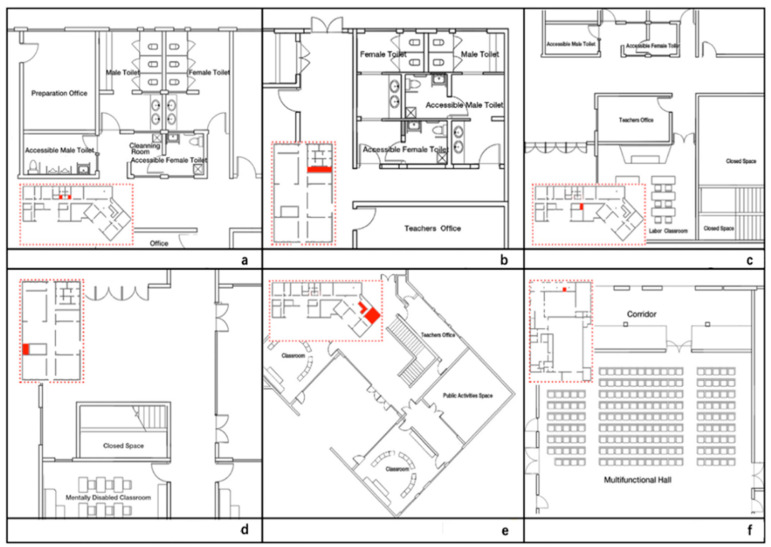
The distribution of the lowest VE areas in the public environment of the school.

In Figure 4, it is demonstrated that the crossed corridors creating spatial diversity for the children have a higher VE. This means that when the children play in the corridors, they can be monitored by their peers or teachers and are safe.

There areas in the public environment of the Deyang primary school with the lowest VE are as follows:

- The linear space as a branch of the main interior corridor, such as the line space in front of the toilets in the Deyi Building and the Dehui Building, and the linear space before entering the labor classroom in the Deyi Building (Figure 4a–c);
- The roads to the rooms or other public spaces behind the elevators, such as the space behind the elevators in the Dehui Building and the Deyin Building (Figure 4d–e);
- The nonlinear public environment with corners that are too deep, such as the public activities room in the Deyin Building (Figure 4e);
- The narrow path connecting the outdoor horizontal corridor and the Sports Center (Figure 4f).

It is suggested that, for deaf children and children with an intellectual disability, whose lives are quite reliant on eye sight, in an area with low VE, strategies to increase the students’ VE towards their peers or professional teachers, such as installing monitors or encouraging the supervisor to pay more attention to those areas, should be deployed to improve the school’s safety and public health.

### 3.2. Social Psychosocial Features in the School Environment

Furthermore, through comparing the spatial position of children in the public school environment and the corresponding values of the VA and VE at these spatial positions, and combining the social psychosocial information reflected from the VA and VE, the authors investigate the social psychosocial features for deaf children or children with an intellectual disability. Two connected buildings, the Deyin and Deyi Buildings, were analyzed.

**Figure 5:**
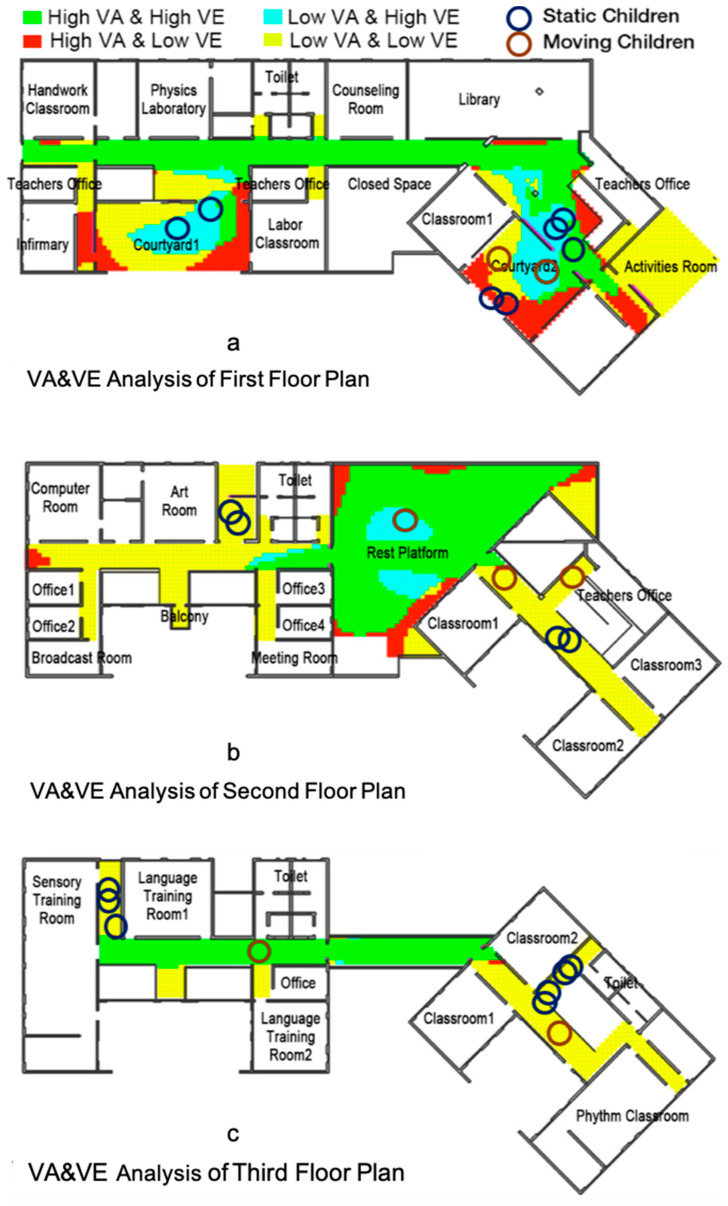
The VA and VE situation and the children’s spatial positions in the Deyin and Deyi Buildings: (**a**) first floor, (**b**) second floor, (**c**) third floor.

Figure 5 illustrates the VE and VA situation of the Deyin and Deyi Buildings from the first floor to the third floor and the spatial positions of the children in the public school environment. Every static child is represented by a blue circle in the public environment and every moving child is circled in red.

By comparing the spatial positions of the children with the VA and VE distribution, it is clear that the VA value is important when children are choosing a place to stay (Table 1). More precisely, children prefer to stay in a place with a low VA (yellow areas and blue areas), such as the middle of the courtyards and the short interior corridors in the Deyin and Deyi Buildings. This means that this kind of child may have more introverted psychological features in the social community. The outcomes are consistent with a few previous scholars [41,42], who stated that, due to their lower language capacity and lack of contact with the outside world when compared to their peers, the psychological features of deaf students are defined as loneliness, lack of confidence, emotional instability, irritability and being introverted. Thus, regarding a public environment with a low VA, in which they prefer to stay, increasing the VE is more important to ensure the safety of deaf children and children with an intellectual disability.

## 4. Discussion

Based on the research results, the authors propose the use of the VAE model as a method to evaluate the physical safety (from the perspective of visual surveillance by their teachers or other people) of the public environment and to indicate the social psychological health features of deaf children and children with an intellectual disability by observing the spatial location of children according to Archea‘s visual analysis method [3,4,5]. These results could reflect the effectiveness of the VAE model methodology in measuring the influence of public environments, especially built architecture, on humans’ physical behavior and public social psychological health status [6,7,8,19].

This research focused on a special group of children who are more sensitive and reliant on the public environment’s safety and visual experience, while psychological health features may be much more crucial for them than for ordinary children, as described by Zhu and Aslam et al. [27,43]. The research design, process and results are primarily based on the scope of Woolcock, Gleeson and Randolph, who stressed the importance of paying attention to children’s health, safety and feelings in relation to the architectural environment [2]. The methodology innovation of piloting the VAE model that Archea proposed [3,4,5] in investigating the public environment’s physical safety and space-relevant public psychological health has been proven to be effective for a group of disabled children in a special school. Moreover, through observing the spaces where deaf children and children with an intellectual disability prefer to stop and by checking the VA and VE features of those spaces, the research also strongly indicates that the spatial configuration of the built environment can infer human behavioral patterns [6,7,8]. In this way, the built architecture could be optimized to improve public health environments.

There are some limitations in this study. Firstly, in the VAE model, every single point in the building plan represents one person, but in real life, the distribution of the population that uses these spaces is unequal. Secondly, the current VAE model lacks efficient consideration of vertical eyesight, and all the VA and VE values are calculated based on the horizontal level. Thirdly, other highly important attributes that can cause differences in people’s behavior were not considered, such as social and cultural factors. The findings in this study may, thus, be either valid or invalid for other cultural communities. Fourthly, due to the time and school limitation of the current study, a large sample was not investigated. Fifthly, there exist a few differences between deaf children and children with intellectual impairments in terms of safety requirements and psychological features, while, in this study, the differences in spatial design were not considered. Sixthly, the authors only observed for one day, and therefore, the study’s results are based only on a single data point.

Further research will be conducted on the following aspects. Firstly, the VAE model will be used for more indoor architectural spaces in different cultural contexts to expand the exploration of the associations between architectural space and behaviors in more human-oriented architecture designs. In addition, it is expected that massive samples will be collected by a long-term field study or advanced sensor technologies in different schools to lead to a more accurate and reliable analysis in the future.

## 5. Conclusions

In this study, the authors suggested using the VAE model as an approach to evaluate the physical safety and social psychological features of the public architectural space for deaf children and children with an intellectual disability. This special children’s group has a stronger visual dependence than normal children; thus, the VAE model is imperative for their safety in public school spaces when compared to their peers. This approach can also be used to evaluate the school architecture of various schools.

According to the study, the potential safety risks of the Deyang children primary school exist in the areas with low VE. The public psychological health features of this children’s group include them being more introverted in the social community, which is reflected in the spatial perspective, and they prefer to stay in places with low VA. The potential applications of this research include the following aspects: firstly, the VAE model can be used as a method to evaluate the schools’ architecture design in terms of safety. With the increasing design projects of schools, such as to enhance the public space inside the building to make a more livable and natural space, the visual access and exposure should be firstly checked to ensure spatial safety. Long corridors and blind spots behind stairs should be avoided in the design. Secondly, an evaluation of the VA and VE through the VAE model could also be conducted for school safety monitoring and management. With the increase in school violence and bullying, strategies to enhance the students’ VE to the teachers and managers should be conducted. Thirdly, this method can be used to determine the students who suffer from psychological health problems from a social community perspective by determining the VA and VE values of a public school space by investigating where various students prefer to stay. This would be positive for their academic merits and psychological health and, in the future, would be beneficial for broader public environmental optimization and public health construction.

## Figures and Tables

**Table 1:** Comparison of the spatial positions of children with the VA and VE distribution.

Number of Children	VA Value	VE Value	Color
2	high VA	high VE	Green
2	high VA	low VE	Red
6	low VA	high VE	Blue
15	low VA	low VE	Yellow

## Data Availability

Data is contained within the article. The data presented in this study are available in Table 1, Figure 3, Figure 4 and Figure 5 in this paper.
